# Novel Discotic Boroxines: Synthesis and Mesomorphic Properties

**DOI:** 10.3390/ma7054045

**Published:** 2014-05-22

**Authors:** Tobias Wöhrle, Angelika Baro, Sabine Laschat

**Affiliations:** Institut für Organische Chemie, Universität Stuttgart, Pfaffenwaldring 55, Stuttgart D-70569, Germany; E-Mails: tobias.woehrle@oc.uni-stuttgart.de (T.W.); angelika.baro@oc.uni-stuttgart.de (A.B.)

**Keywords:** boroxines, liquid crystals, columnar, synthesis

## Abstract

A new synthetic approach to highly substituted triphenylboroxines **11** is described. Their mesomorphic properties were investigated by differential scanning calorimetry (DSC), polarizing optical microscopy (POM) and X-ray diffraction (SAXS, WAXS). The tris(3,4,5-trialkyloxy)phenyl functionalized derivatives **11b**–**e** showed broad mesophases for a minimum alkyl chain length of C9. The phase widths ranged from 110 K to 77 K near room temperature, thus decreasing with enhanced alkyl chain lengths. Textures observed under POM indicated a columnar hexagonal (Col_h_) mesophase symmetry that was confirmed by X-ray diffraction experiments.

## 1. Introduction

The discovery of stable liquid crystalline phases formed by disk-shaped molecules with long alkyl chains in their periphery is generally dated to the seminal work of Chandrasekar published in 1977 [1]. Since then discotic liquid crystals have attracted the attention of many research groups worldwide [2–4]. Due to the one dimensional charge and ion transport in the columnar mesophase, the ability of liquid crystals (LCs) to self-heal structural defects by thermal annealing and the ease of processing via spin coating, drop casting and other solution processing methods highly useful applications [5–7] could berealized such as organic solar cells [8], organic field effect transistors [3] and organic light emitting diodes [9].

**Figure 1.**
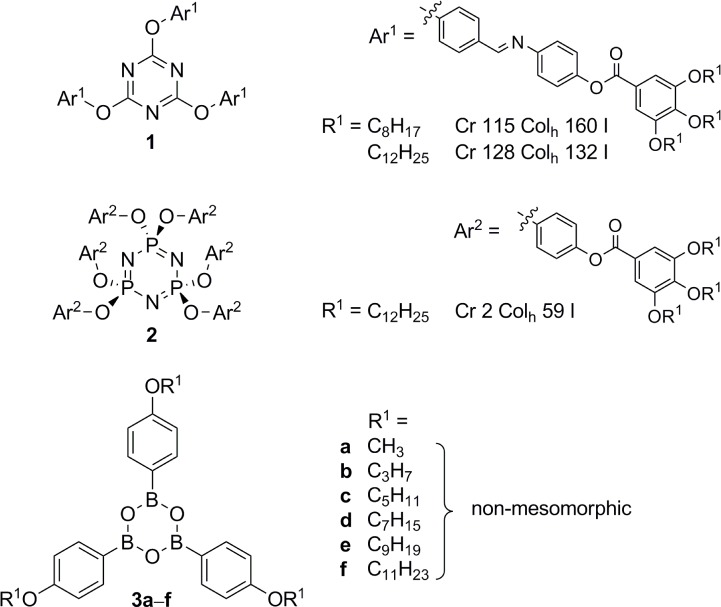
Chemical structure of liquid crystals (LC) materials containing a heterocyclic six-membered ring as core unit.

From a molecular perspective, several scaffolds have turned out to be successful candidates for the nanosegregation, which ultimately led into the formation of columnar mesophases. Besides tri- and hexasubstituted benzenes, in particular triphenylenes [10], perylenes [11], hexa-*peri*-hexabenzocoronenes [12,13], porphyrins [14,15], phthalocyanines [15], quinoxalines and other aza analogues of polycyclic aromatic hydrocarbons were used as mesogenic subunits. However, not only disk-shaped mesogens, but also non-conventional [16] and supramolecular and hydrogen bonded liquid crystals [17] can self-assemble into columnar aggregates. While a variety of LC materials containing heterocyclic 6-membered rings have been synthesized [18], e.g., triazines such as **1** [19–21] or cyclotri­phosphazenes such as **2** [22,23] (Figure 1), surprisingly little is known about liquid crystalline boroxines, the cyclic trimers of organoboronic acids. Aryl boronic acids are valuable reagents for a number of metal-catalyzed reactions, the most prominent one thereof is certainly the Suzuki-Miyaura cross-coupling reaction [24–28]. Furthermore, boroxines may serve as useful building blocks for flame retardants, lithium ion battery materials and covalent organic frameworks [29,30]. The only example considering mesomorphism was a report by Preece and coworkers who studied a series of tris(alkoxyphenyl)boroxines **3**, which, however, did not reveal any mesophases (Figure 1) [31].

Based on this precedence we anticipated that the attachment of additional alkoxy groups at the aryl rings should favor nanosegregation and thus induce mesophase formation. The results towards this goal are reported below.

## 2. Results and Discussion

### 2.1. Synthesis of Boroxines

First, we intended to attach alkyloxy groups with chains lengths of C12-C18 at the phenyl rings in boroxine **3** following known procedures [32–35]. However, only **3g** (R^1^ = C_12_H_25_) could be isolated in 30% yield, whereas boroxines with alkyl chain lengths >12 were not obtained as a problem of poor solubility of the precursor boronic acid (for details see Supplementary Information). Derivative **3g** did not show mesomorphic properties.

**Scheme 1.**
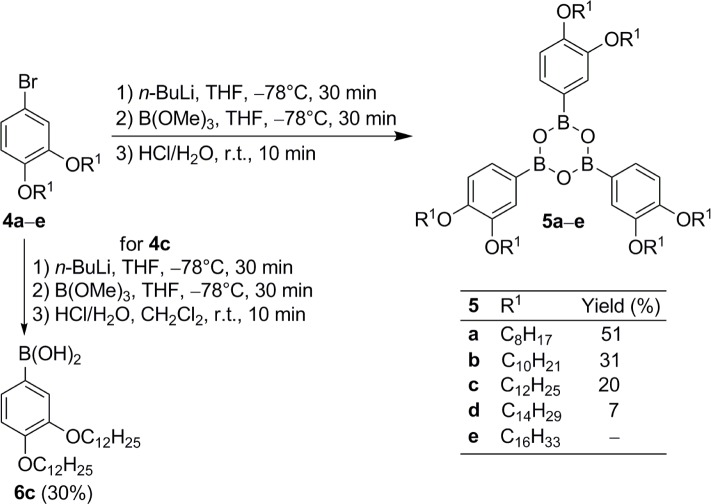
Synthesis of boroxines **5** starting from the respective dialkoxy-bromobenzenes **4**. Boronic acid **6c** was prepared for comparison and assignment of ^1^H NMR and IR data (see Supplementary Information).

Following our envisioned strategy, 4-bromo-1,2-dialkoxybenzenes **4** [36,37] were lithiated with *n*-BuLi, THF at −78 °C, followed by treatment with B(OMe)_3_ and then directly hydrolyzed by addition of aqueous HCl to the mixture. No trace of the boronic acids was detected, but the boroxines **5** were isolated (Scheme 1). Their yields decreased considerably with increasing chain lengths from 51% for **5a** to 7% for **5d** due to purification problems. Column chromatography led to decomposition, thus leaving recrystallization as only method to purify **5**. With increasing chain length the solubility of dialkoxybromides **4**, boronic acids **6** and boroxines **5** was more and more alike. Therefore, the crude product of **5e** with two C_16_H_33_ groups at the phenyl ring was found as an inseparable mixture of those three and no pure product could be obtained.

**Scheme 2.**
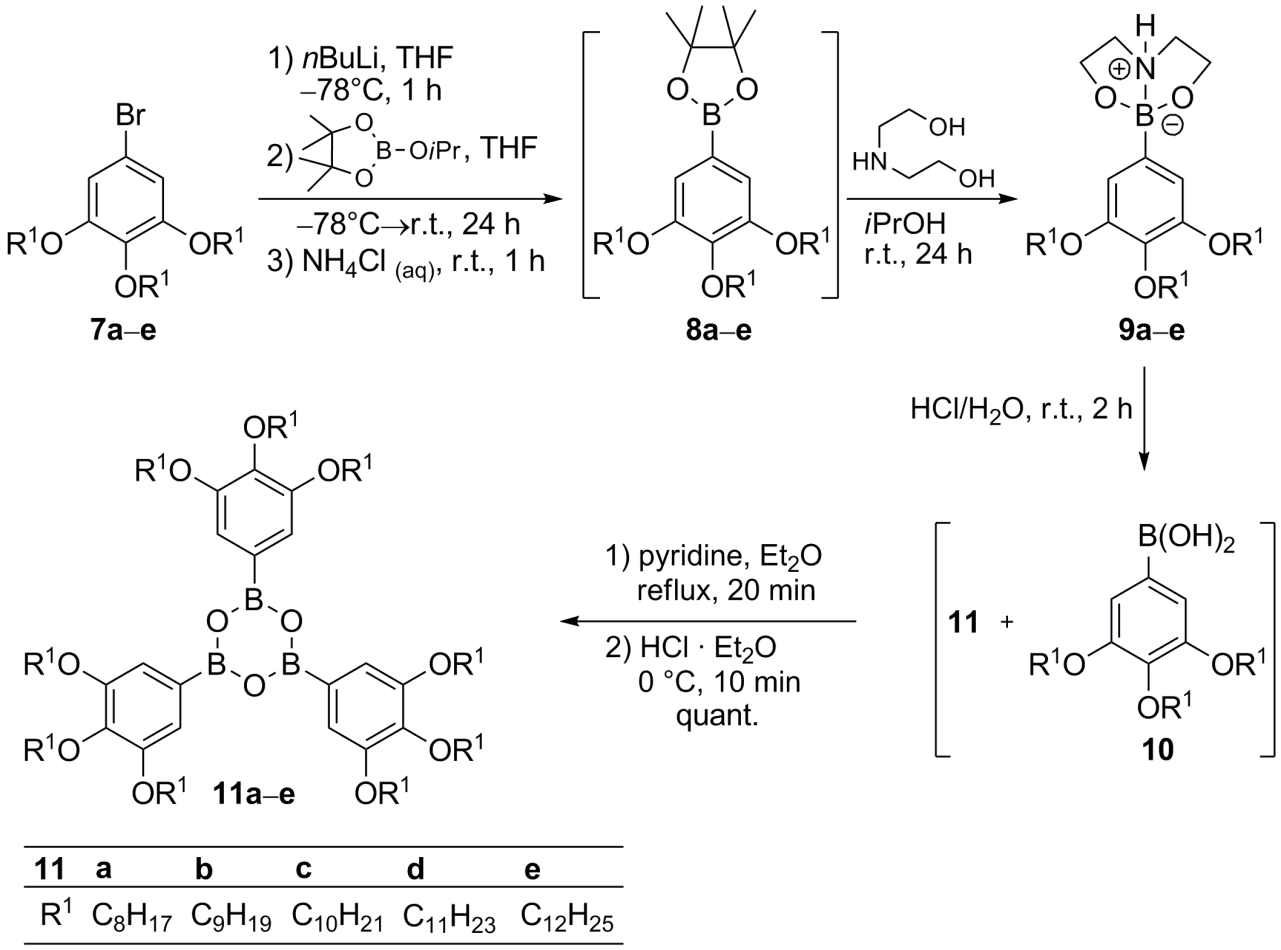
Synthesis of boroxines **11a**–**e** bearing three 3,4,5-trialkoxyphenyl substituents. For comparison, diethanolamine complex **9e** was isolated. Its ^1^H and ^13^C NMR spectra are given together with characteristic IR bands for distinction of **10** and **11** (Table S2 in the Supplementary Information).

The synthesis of tris(3,4,5-trialkoxyphenyl)boroxines **11** commenced with the 5-bromo-1,3,4-trialkoxybenzenes **7** [38–41]. Their conversion into the boronic acids according to the method described in Scheme 1 turned out to be problematic, because inseparable mixtures of boronic acids and boroxines were obtained. This was also true, when the initial bromo-lithium exchange was replaced by formation of a Grignard species instead. Therefore, an alternative method was applied (Scheme 2).

Bromides **7** were treated with *n*-BuLi, followed by addition of isopropoxypinacolborolane [42] to yield the corresponding pinacolborolanes **8** [38] which were directly treated with bisethanolamine in *i*-PrOH. The resulting diethanolamine complexes **9** precipitated from the solution and after filtration, they were hydrolyzed without further purification with HCl in THF giving a mixture of boroxines **11** and boronic acids **10**. Refluxing this mixture with pyridine in Et_2_O [43] followed by hydrolysis with HCl in Et_2_O provided the desired free boroxines **11** quantitatively. Noteworthy, this strategy did not need purification at any step. Only pinacolborolanes **8** were deprotected to complexes **9** which precipitated in analytically pure form (shown for **9e** in the Supplementary Information). The subsequent two reaction steps proceeded in quantitative yield without any impurities. The overall yields of **11** starting from **7** were in the range of 20%–40%. The solid products **11** were air-stable, however, storage in solvents containing traces of water led to slow decomposition to the corresponding boronic acids **10**.

### 2.2. Mesomorphic Properties of Boroxines

The liquid crystalline properties of compounds **5** and **11** were studied by differential scanning calorimetry (DSC), polarizing optical microscopy (POM) and X-ray diffraction (XRD: wide-angle X-ray scattering (WAXS), small-angle X-ray scattering (SAXS)).

**Figure 2.**
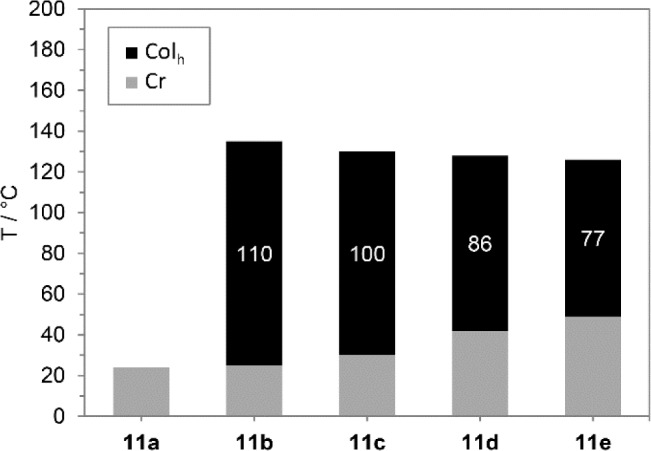
Mesophase stabilities of boroxines **11a**–**e** upon second heating (heating rate 5 K·min^−1^). Mesophase widths are given.

All compounds **5a**–**d** bearing three 3,4-dialkoxyphenyl substituents did not show any mesomorphism. In contrast, boroxines **11** with additional alkoxy group turned out to be mesogenic with exception of derivative **11a** with C8 alkyloxy side chains. The results obtained from DSC measurements are summarized in Table 1 and Figure 2.

Boroxines **11b**–**e** displayed broad mesophases near room temperature. For boroxine **11b** with C9 alkyloxy side chains a liquid crystalline phase between 25 °C and 135 °C was observed. With increasing chain lengths melting points continuously rose up to 49 °C (**11e**) and clearing points receded to 123 °C resulting in reduced phase widths from 100 K (**11c**) to 77 K (**11e**).

**Figure 3.**
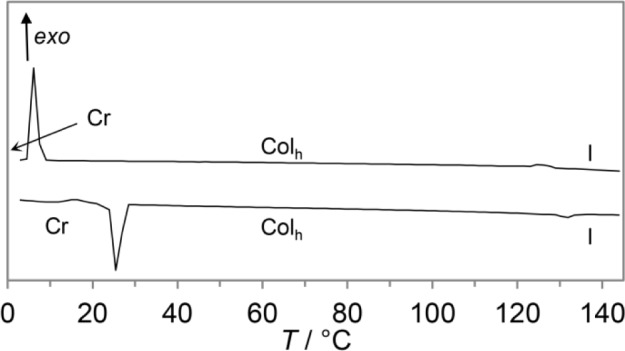
DSC curves of **11b** (second heating/cooling cycle, heating/cooling rate 5 K·min^−1^).

Considering the DSC traces of **11b**–**11e** identical behaviour of all derivatives during heating and cooling cycle is evident. As example, DSC curves of **11e** are shown in Figure 3 (for further DSC curves see Supplementary Information). Melting points were clearly visible while clearing point peaks were less intensive and very broad. Hence, peak temperatures are given in Table 1. Upon cooling both melting and clearing point displayed a supercooling of phase transitions. Clearing points were slightly affected. Boroxine **11b** shows the largest shift of 6 K from 135 °C upon heating to 129 °C upon cooling. The melting points of **11b** and **11d**, however, were significantly shifted by 18 K from 25 °C (**11b**) and 42 °C (**11d**) to 7 °C and 24 °C, respectively.

**Figure 4.**
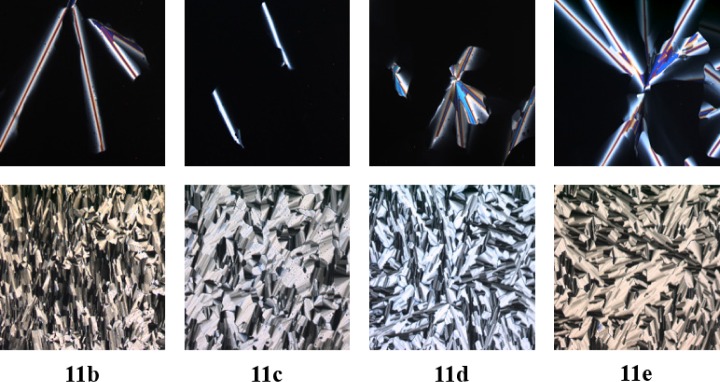
Textures of **11b**–**e** as seen between crossed polarizers upon cooling from isotropic liquid (cooling rate 5 K·min^−1^, magnification ×100). (**top**): standard glass slides, (**bottom**): trimethylchlorosilane-treated glass slides.

Under the microscope boroxines **11b**–**11e** behaved similarly. Upon cooling from the isotropic liquid all derivatives formed homeotropic areas with few defects (Figure 4 top).

Presumably, the boron atoms coordinate at the oxygen atoms of the glass surface, resulting in aligned mesophases perpendicular to the surface. Therefore, the glass slides were treated with trimethylchlorosilane prior to use. In this way, broken fan-shaped textures being typical for columnar mesophases could be observed for boroxines **11b**–**11e** under the POM (Figure 4 bottom).

**Figure 5.**
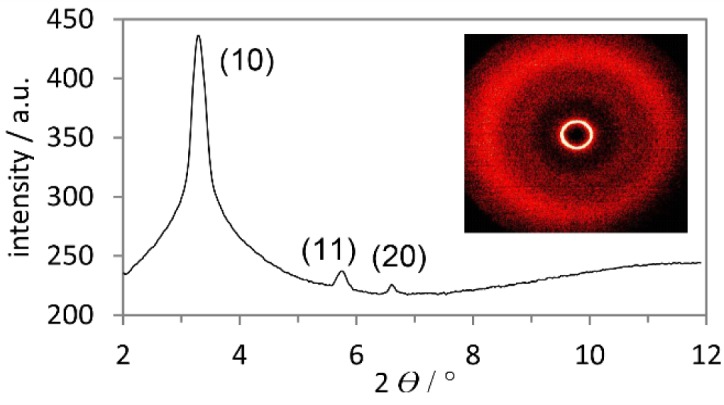
Small-angle X-ray scattering (SAXS) profile of the liquid-crystalline phase of **11e** at 100 °C (Inset: wide-angle X-ray scattering, WAXS).

In order to get further insight into the phase geometry, derivatives **11b**–**e** were investigated by XRD (SAXS and WAXS). Figure 5 shows both the SAXS profile and WAXS pattern exemplarily for boroxine **11e**. In the small-angle region three reflections are visible in a ratio of 1:1/ $\sqrt{3}$:1/2 which were indexed as (10), (11) and (20) [44]. This diffraction pattern is typical for columnar hexagonal (Col_h_) phase geometries. In the wide-angle region a diffuse halo was observed, which is generated through the interaction of the molten-like alkyl chains. In the case of derivatives **11b**–**d**, however, only the (10) reflection and the diffuse halo were clearly visible in the diffractograms. Due to identical molecule geometry also Col_h_ mesophases are assumed for **11b**–**d** (for XRD data see Supplementary Information).

## 3. Experimental Section

### 3.1. Materials

All reagents were used as purchased from the suppliers without further purification. Solvents were dried and distilled under nitrogen prior to use and unless otherwise stated all reactions were carried out under nitrogen atmosphere with Schlenk-type glassware.

### 3.2. General Procedure for the Synthesis of Tris(3,4,5-Trialkoxyphenyl)boroxines (**11**)

To a solution of the appropriate **7** (4.225 mmol) in abs. THF (150 mL) at −78 °C was added *n*-BuLi (4.50 mL, 7.2 mmol, 1.6 M in *n*-hexane, Merck KGaA, Darmstadt, Germany) and the reaction mixture was stirred for 1 h. Then isopropyl pinacol borate (1.32 mL, 1.20 g, 6.34 mmol, Sigma-Aldrich, Steinheim, Germany) was added and the reaction mixture stirred for a further 1 h at −78 °C. After warming to room temperature over 24 h, the reaction was terminated by addition of NH_4_Cl (50 mL, saturated solution) and stirring for 1 h. The resulting aqueous suspension was extracted with Et_2_O (3 × 50 mL). The combined organic layers were washed with H_2_O (2 × 100 mL) and brine (80 mL) and dried (MgSO_4_). The solvent was removed under vacuum and the crude product **8** was directly used for the next step.

The crude appropriate pinacolborolane **8** was dissolved in a minimal amount of isopropanol, diethanolamine (0.85 mL, 0.89 g, 8.45 mmol, calcd for quantitative yield in the previous step, Sigma-Aldrich, Steinheim, Germany) was added, and the mixture was stirred for 24 h at ambient temperature. The suspension was filtered giving the boronic acid diethanolamine complexes **9** as white solids. Those were dissolved in THF (15 mL) and stirred with HCl (7 mL, 2 M) for 2 h at room temperature. The resulting aqueous suspension was extracted with Et_2_O (3 × 30 mL). The combined organic layers were washed with H_2_O (2 × 500 mL) and brine (50 mL) and dried (MgSO_4_). The solvent was removed under vacuum giving mixtures of boronic acids **10** and the appropriate boroxines **11** in good overall yield.

The respective mixture of **10** and **11** (0.3 mmol, calcd for the maximum amount of **10**) was dissolved in abs. Et_2_O and, together with mol sieves (4 Å) to trap resulting water, heated to reflux. Pyridine (abs., 0.1 mL, 0.095 g, 1.2 mmol) was added and the mixture was stirred for additional 20 min. The solution was then cooled with ice (0 °C) and HCl·Et_2_O (pH = 1) was added. After 10 min, the precipitate was collected on a glass fritted funnel and the filtrate was fully evaporated *in vacuo* to give the desired boroxines **11** as colorless solids in quantitative yield.

#### 3.2.1. Tris(3,4,5-Trioctyloxyphenyl)boroxine (**11a**)

^1^H NMR (500 MHz, CDCl_3_): δ = 0.83–0.95 (m, 27H, CH_3_), 1.23–1.43 (m, 72H, CH_2_), 1.44–1.56 (m, 18H, OCH_2_CH_2_CH_2_), 1.72–1.90 (m, 18H, OCH_2_CH_2_), 4.01–4.14 (m, 18H, OCH_2_), 7.39 (s, 6H, 2-H) ppm. ^13^C NMR (126 MHz, CDCl_3_): δ = 14.11, 14.12 (CH_3_), 22.70, 22.72, 26.1, 26.2, 29.3, 29.40, 29.46, 29.55, 29.56, 30.39, 31.87, 31.93 (CH_2_), 69.2, 73.5 (OCH_2_), 113.9 (C-2), 142.7 (C-4), 152.9 (C-3) ppm. FT-IR (ATR): $\tilde{v}$ = 2927 (w), 2855 (w), 1574 (w), 1467 (w), 1417 (w), 1333 (s, B-O), 1110 (w), 904 (s), 726 (vs, BX), 649 (s) cm^−1^ (BX denotes the anhydride band [45]). MS (MALDI-TOF): *m*/*z* calcd. for [C_90_H_159_B_3_O_12_]^+^ 1465.21, found 1465.04. Anal. calcd. for C_90_H_159_B_3_O_12_ (1465.68 g·mol^−1^): C 73.75, H 10.93; found: C 73.39, H 11.02.

#### 3.2.2. Tris(3,4,5-Trinonyloxyphenyl)boroxine (**11b**)

^1^H NMR (500 MHz, CDCl_3_): δ = 0.85–0.91 (m, 27H, CH_3_), 1.21–1.43 (m, 90H, CH_2_), 1.45–1.56 (m, 18H, OCH_2_CH_2_CH_2_), 1.73–1.90 (m, 18H, OCH_2_CH_2_), 4.04–4.13 (m, 18H, OCH_2_), 7.39 (s, 6H, 2-H) ppm. ^13^C NMR (126 MHz, CDCl_3_): δ = 14.12 14.13 (CH_3_), 22.71, 22.72, 26.1, 26.2, 29.3, 29.4, 29.52, 29.55, 29.62, 29.66, 29.7, 30.4, 31.94, 31.97 (CH_2_), 69.2, 73.5 (OCH_2_), 113.9 (C-2), 142.7 (C-4), 152.9 (C-3) ppm. FT-IR (ATR): $\tilde{v}$ = 2958 (w), 2924 (w), 2853 (w), 1575 (w), 1467 (w), 1417 (w), 1334 (s, B-O), 1261 (w), 1213 (w), 1111 (w), 1021 (w), 905 (s), 849 (w), 804 (w), 725 (vs, BX), 649 (w) cm^−1^. MS (MALDI-TOF): *m*/*z* calcd. for [C_99_H_177_B_3_O_12_]^+^ 1591.35, found 1591.79. Anal. calcd. for C_99_H_177_B_3_O_12_ (1591.92 g·mol^−1^): C 74.70, H 11.21; found: C 74.30, H 11.30.

#### 3.2.3. Tris(3,4,5-Tridecyloxyphenyl)boroxine (**11c**)

^1^H NMR (500 MHz, CDCl_3_): δ = 0.82–0.96 (m, 27H, CH_3_), 1.17–1.45 (m, 108H, CH_2_), 1.45–1.63 (m, 18H, OCH_2_CH_2_CH_2_), 1.71–1.93 (m, 18H, OCH_2_CH_2_), 4.02–4.13 (m, 18H, OCH_2_), 7.39 (s, 6H, 2-H) ppm. ^13^C NMR (126 MHz, CDCl_3_): δ = 14.1 (CH_3_), 22.7, 26.1, 26.2, 29.40, 29.41, 29.51, 29.54, 29.60, 29.65, 29.7, 29.8, 30.38, 31.93, 31.95 (CH_2_), 69.22, 73.50 (OCH_2_), 113.9 (C-2), 142.7 (C-4), 152.9 (C-3) ppm. FT-IR (ATR): $\tilde{v}$ = 2958 (w), 2923 (w), 2853 (w), 1574 (w), 1466 (w), 1417 (w), 1333 (s, B-O), 1261 (w), 1212 (w), 1112 (w), 1014 (w), 905 (s), 801 (w), 723 (vs, BX), 645 (w) cm^−1^. MS (MALDI-TOF): *m*/*z* calcd. for [C_108_H_195_B_3_O_12_]^+^ 1717.49, found 1717.89. Anal. calcd. for C_108_H_195_B_3_O_12_ (1718.16 g·mol^−1^): C 75.50, H 11.44; found: C 75.25, H 11.46.

#### 3.2.4. Tris(3,4,5-Triundecyloxyphenyl)boroxine (**11d**)

^1^H NMR (500 MHz, CDCl_3_): δ = 0.80–0.94 (m, 27H, CH_3_), 1.20–1.43 (m, 126H, CH_2_), 1.45–1.58 (m, 18H, OCH_2_CH_2_CH_2_), 1.70–1.93 (m, 18H, OCH_2_CH_2_), 4.02–4.13 (m, 18H, OCH_2_), 7.39 (s, 6H, 2-C) ppm. ^13^C NMR (126 MHz, CDCl_3_): δ = 14.1 (CH_3_), 22.7, 26.1, 26.2, 29.4, 29.50, 29.54, 29.6, 29.71, 29.75, 29.76, 30.4, 31.94, 31.96 (CH_2_), 69.2, 73.50 (OCH_2_), 113.9 (C-2), 142.7 (C-4), 152.9 (C-5) ppm. FT-IR (ATR): $\tilde{v}$ = 2958 (w), 2920 (s), 2851 (w), 1575 (w), 1506 (w), 1467 (w), 1419 (s), 1336 (vs, B-O), 1261 (w), 1241 (w), 1212 (w), 1172 (w), 1111 (s), 1021 (w), 845 (w), 802 (w), 721 (s, BX), 667 (w), 648 (w) cm^−1^. MS (MALDI-TOF): *m*/*z* calcd. for [C_117_H_213_B_3_O_12_]^+^ 1843.63, found 1843.49. Anal. calcd. for C_117_H_213_B_3_O_12_ (1844.41 g·mol^−1^): C 76.19, H 11.64; found: C 76.39, H 11.33.

#### 3.2.5. Tris(3,4,5-Tridodecyloxyphenyl)boroxine (**11e**)

^1^H NMR (500 MHz, CDCl_3_): δ = 0.88 (m, 27H, CH_3_), 1.17–1.44 (m, 144H, CH_2_), 1.45–1.61 (m, 18H, OCH_2_CH_2_CH_2_), 1.74–1.90 (m, 18H, OCH_2_CH_2_), 4.00–4.16 (m, 18H, OCH_2_), 7.39 (s, 6H, 2-H) ppm. ^13^C NMR (126 MHz, CDCl_3_): δ = 14.12 (CH_3_), 22.7, 26.1, 26.2, 29.4, 29.53, 29.55, 29.6, 29.72, 29.77, 29.78, 30.4, 31.9 (CH_2_), 69.23, 73.50 (OCH_2_), 113.9 (C-2), 142.7 (C-4), 152.9 (C-3) ppm. FT-IR (ATR): $\tilde{v}$ = 2957 (w), 2919 (s), 2850 (s), 1575 (w), 1467 (w), 1419 (s), 1336 (vs, B-O), 1260 (w), 1213 (w), 1113 (s), 1012 (w), 907 (w), 846 (w), 799 (w), 736 (s), 720 (s, BX), 648 (w), 578 (w) cm^−1^. MS (MALDI-TOF): *m*/*z* calcd. for [C_126_H_231_B_3_O_12_-H]^+^ 1969.77, found 1968.80. Anal. calcd. for C_126_H_231_B_3_O_12_ (1970.65 g·mol^−1^): C 76.80, H 11.82; found: C 77.02, H 11.92.

### 3.3. Instrumental Analysis

The following instruments were used for characterization of the compounds. NMR: Bruker Avance 500 (^1^H, 500 MHz; ^13^C, 126 MHz) and Bruker Avance 300 (^1^H, 300 MHz; ^13^C, 75 MHz). ^1^H and ^13^C NMR spectra were referenced to tetramethylsilane (TMS, Sigma-Aldrich, Steinheim, Germany) (δ_H_ = 0.0 ppm, δ_C_ = 0.0 ppm) as an internal standard. Unless otherwise stated, spectra were recorded at room temperature. Assignment of the resonances was supported by chemical shift calculations and 2D experiments (COSY and HMBC). Elemental analyses: Carlo Erba Strumentazione Elemental Analyzer, Modell 1106. IR: Bruker Vector 22 FT-IR Spectrometer with MKII golden gate single reflection Diamant ATR system. MS (ESI): Bruker Daltonics microTOF-Q spectrometer. Polarizing optical microscopy: Olympus BX50 polarizing microscope combined with a Linkam TP93 central controller. X-ray diffraction (WAXS, SAXS regions): Bruker AXS Nanostar C diffractometer employing Ni-filtered CuKα radiation (λ = 1.5418 Å), standard stationary temperature control unit for temperature programs. Differential scanning calorimetry (DSC): Mettler-Toledo DSC 822e. Flash chromatography was performed on silica gel, grain size 40–63 μm (Fluka) and aluminium sheets precoated with silica gel 60 F254 (Merck) were used for thin layer chromatography.

## 4. Conclusions

A new and easy approach towards discotic boroxines and study of their mesomorphic properties is reported. Pure boroxines **11** were accessible starting from 5-bromo-1,3,4-trialkoxybenzenes **7** via four-step reaction which did not need purification of intermediates.

Derivatives **5** bearing three dialkyloxyphenyl groups are found to be non-mesomorphic. In contrast, linkage of three trialkyloxyphenyl substituents gave boroxines **11** displaying liquid crystalline behaviour. A minimum of C9 alkyl chain length (**11b**) turned out to be necessary for mesophase formation because **11a** with C8 alkyl chains is non-mesogenic. The phase widths ranged from 77 K (**11e**) to 110 K (**11b**) near room temperature, thus decreasing with enhanced alkyl chain lengths. Under POM, fan-shaped textures typical for columnar mesophases were observed for all boroxines **11b**–**e**. The phase geometry was further supported by X-ray diffraction showing in the small-angle region the typical diffraction pattern of columnar hexagonal mesophases.

## Figures and Tables

**Table 1.** Phase transitions of the mesogenic boroxines **11a**–**e**.^a,b^

Compound	Phase	*T*_m_ [°C] (Δ*H* [kJ·mol^−1^])	Phase	*T*_c_ [°C] (Δ*H* [kJ·mol^−1^])	Phase	Cycles
**11a**	Cr	24	–	–	I	2nd heating
**11b**	Cr	25 (65.5)	Col_h_	135 ^c^ (3.4)	I	2nd heating
**11b**	Cr	7 (25.2)	Col_h_	129 ^c^ (1.3)	I	2nd cooling
**11c**	Cr	30 (84.3)	Col_h_	130 ^c^ (7.7)	I	2nd heating
**11c**	Cr	22 (73.4)	Col_h_	127 ^c^ (4.4)	I	2nd cooling
**11d**	Cr	42 (116.4)	Col_h_	128 ^c^ (2.3)	I	2nd heating
**11d**	Cr	24 (72.7)	Col_h_	126 (2.5)	I	2nd cooling
**11e**	Cr	49 (123.9)	Col_h_	126 ^c^ (5.6)	I	2nd heating
**11e**	Cr	33 (86.1)	Col_h_	123 (3.3)	I	2nd cooling

a The following phases were observed: crystalline (Cr), columnar hexagonal (Col_h_), isotropic liquid (I)

b transition temperatures were determined by DSC (heating/cooling rate 5 K·min^−1^)

c peak temperature.

## Supplementary Materials

Supplementary File 1
