# Supplementary material for: Novel Discotic Boroxines: Synthesis and Mesomorphic Properties
Source: Materials (Basel). 2014 May 22;7(5):4045–56. doi: 10.3390/ma7054045 (PMC5453236; doi:10.3390/ma7054045)
Supplement: Supplementary File 1 [file materials-07-04045-s001.pdf]

Supplementary Information

2. Results and Discussion

2.1. Synthesis of Boroxines

The *p*-alkoxybromobenzenes **12**, which were obtained by Williamson etherification according to the procedure by An [1–3] were submitted to halogen-lithium exchange with *n*-BuLi, followed by treatment with $B(OMe)_3$ and subsequent acidic hydrolysis providing the boronic acids **13** together with the arylboroxines **3** as byproducts (Scheme S1). In case of the dodecyloxy-substituted derivative, boronic acid **13a** was isolated in only 11% together with 30% of the boroxine **3g** after column chromatography. For longer alkyl chains (R = $C_{14}H_{29}$–$C_{18}H_{37}$) the boroxines **3h–j** were only detected in the crude product but could not be isolated, whereas the isolated yields of boronic acids **13b–d** were still quite low (21%–22%).

Several conditions were tried for the condensation (Table S1). Heating of **13c** in toluene in a Dean-Stark trap for 16 h under reflux (method A) or in the presence of 6 mol% of TsOH under Dean-Stark conditions (method B) resulted in a (1:1:1) mixture of boronic acid **13c**, boroxine **3i** and byproduct, which could not be separated by column chromatography. Condensation of the neat compound **13c** under reduced pressure according to the method by Wu [4] (method C). Again varying mixtures of boronic acid **13c**, boroxine **3i** and byproduct were obtained (entries 3–6). The most promising result is shown in entry 5, but the boroxine **3i** again could not be isolated in pure form.

Scheme S1. Synthesis of boroxines **3g–j**. Boroxines **3h–i** were only detected in the crude product by ^{1}H-NMR.

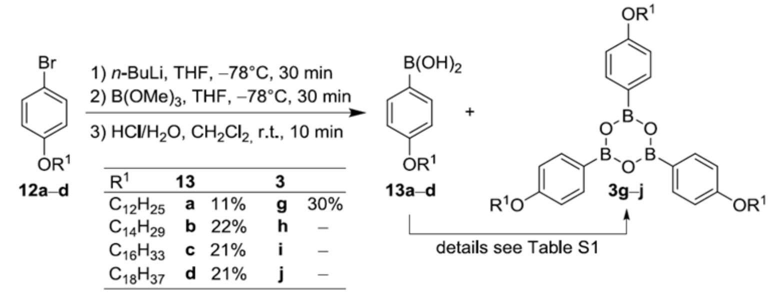

Table S1. Condensation of 4-dodecyloxyphenyl boronic acid **13c** to the corresponding boroxine **3i** under various conditions. [a,b]

Entry	Method	Temperature [°C]	Pressure [mbar]	Time	Product Ratio		
					**3i**	**13c**	**Byprod.**
(1)	A	115	760	16 h	1	1	1
(2)	B	115	760	24 h	1	1	1
(3)	C	110	600	120 min	0.25	1	1
(4)	C	120	700	90 min	0.5	1	0.5
(5)	C	120	700	40 min	0	1	0.5
(6)	C	120	700	17 min	1	0.25	0

[a] Reaction conditions: method A: Dean-Stark trap, toluene, 1.00 mM, 115 °C; method B: 6 mol% TsOH, Dean-Stark trap, toluene, 1.00 mM, 125 °C; method C: neat, heating in a revolver oven. [b] Product ratio was determined by ^{1}H-NMR of the crude product.

2.2. Identification of Boroxines

It should be noted, that boronic acids **13** and boroxines **3** could be easily distinguished by ^{1}H NMR, because the aryl C-H signals are downfield shifted for boroxine **3** as compared to boronic acid **13**. Furthermore boronic acid **13** shows a B-OH signal at 9.15 ppm in DMSO-d_6. If the spectrum was measured in $CDCl_3$, the B-OH signal was upfield shifted to 5.51 ppm. Typical spectra are shown in Figure S1 for **12a**, **13a**, **3g**.

Further evidence came from the IR spectra for **13a**, **3g** (Figure S2). Boronic acid **13a** displays the O-H stretching frequency at 3607 cm^{-1}. The same procedure was used to identify boroxines **5** and **11** hence boronic acids **6c** and **10d** were synthesised, and characteristic IR bands which allow distinguishing between boronic acids and boroxines **3g**, **5b–d**, and **11a–d** are listed in Table S2.

From the compounds listed in Table S2 only the boronic acids **13a**, **6c** and **10d** show the BO-H vibration. The B-O band, which slightly varies from 1320 to 1352 cm^{-1} and is visible for all compounds, is nearly constant in each series of derivatives with identical substitution pattern. A similar trend was observed for the anhydride band BX [5], which was found only in the case of boroxines. Increasing the number of alkyloxy substituents shifted the BX band to larger wave numbers from 688 to 726 cm^{-1}, whereas the alkyl chain lengths in the same substitution pattern has no (or a minor) effect on the absorption. The presence, or absence, of this absorption band strongly indicates the presence, or absence, of a boroxine. Comparable data can be found in a work from Snyder, Konecky and Lennarz [5].

Figure S1. Comparison of chemical shifts of aryl C-H signals for **12a**, **13a** and **3g**. (Spectra of **12a** and **3g** in $CDCl_3$, of **13a** in DMSO-d_6).

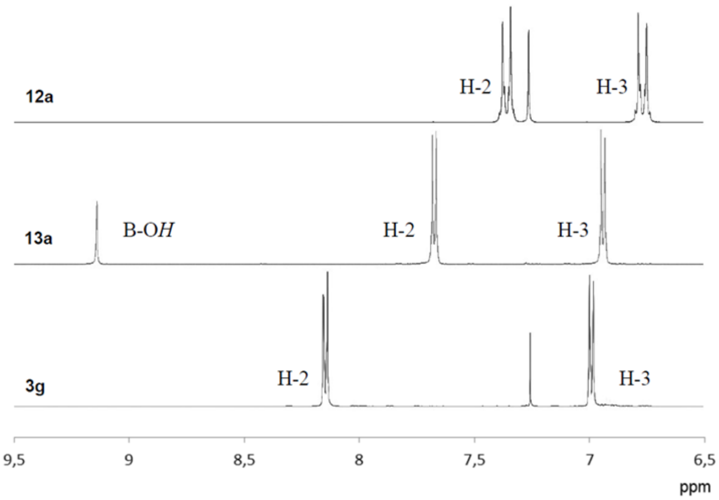

Table S2. Characteristic IR bands of boronic acids **3g**, **6c**, **10d** and the boroxines.

Compound	BO-H Band/cm^{-1}	B-O Band/cm^{-1}	BX Band/cm^{-1}
13a	3607	1320	–
3g	–	1351	688
6c	3453	1334	–
5b	–	1351	710
5c	–	1352	710

Table S2. *Cont.*

Compound	BO-H Band/cm^{-1}	B-O Band/cm^{-1}	BX Band/cm^{-1}
5d	–	1352	710
10d	3437	1337	–
11a	–	1333	726
11b	–	1334	725
11c	–	1333	723
11d	–	1336	721
11e	–	1336	720

Figure S2. Comparison of IR spectra of boronic acid **13a** and boroxine **3g**.

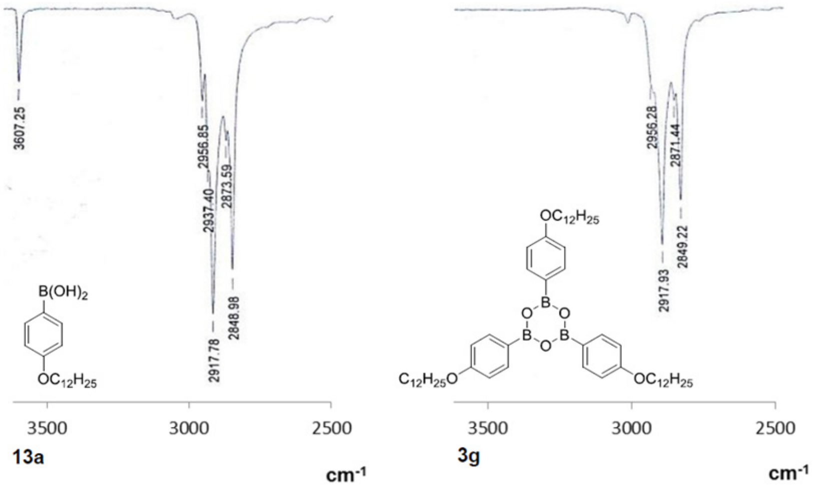

3. Experimental Section

*3.1. General Procedure for the Synthesis of Alkoxyphenylboronic Acids (**13, 6c, 10d**) and Boroxine (**3g**) [6–8]*

A solution of the appropriate **12**, **4**, **7** (5.00 mmol) in abs. THF (20 mL) was added dropwise at −78 °C to a solution of *n*-BuLi (3.38 mL, 5.25 mmol, 1.6 M in *n*-hexane, Merck KGaA, Darmstadt, Germany) and THF (80 mL). After stirring for 30 min, trimethylborate (0.85 mL, 0.78 g, 7.50 mmol, Sigma-Aldrich, Germany) was added, the reaction mixture stirred for a further 30 min at −78 °C and then warmed to room temperature. The solvent was removed under vacuum, the residue taken up in CH_2Cl_2 (50 mL) followed by addition of HCl (25 mL, 2 M Lösung,). After stirring for 10 min at room temperature, the layers were separated. The organic layer was washed with H_2O (2 × 30 mL) and brine (30 mL), dried ($MgSO_4$) and the solvent removed under vacuum. The crude product was purified by column chromatography on SiO_2.

*3.2. 4-Dodecyloxyphenylboronic Acid (**13a**)*

Purification by chromatography on SiO_2 with hexanes/EtOAc (20:1), colorless solid (11%, purity > 95%), m.p. 96 °C, R_f = 0.25. ^{1}H-NMR (500 MHz, DMSO-d$_6$, 110 °C) δ = 0.88 (t, *J* = 6.9 Hz, 3H, CH_3), 1.22–1.50 (m, 18H, CH_2), 1.70–1.79 (m, 2H, OCH_2CH_2), 4.04 (t, *J* = 6.5 Hz, 2H, OCH_2),

6.94 (d, J = 8.6 Hz, 2H, H-3), 7.67 (d, J = 8.6 Hz, 2H, H-2), 9.13 (s, 0.5H, OH) ppm. ^{13}C-NMR (500 MHz, DMSO-d_6, 110 °C) δ = 14.1 (CH_3), 22.4 (CH_2CH_3), 25.9, 29.00, 29.11, 29.22, 29.31, 29.34, 29.36, 31.7 (CH_2), 68.2 (OCH_2), 114.4 (C-3), 136.8 (C-2), 161.2 (C-4). FT-IR (ATR): $\tilde{\nu}$ = 3607.3 (w, BO-H), 2957 (w), 2937 (w), 2918 (s), 2874 (w), 2849 (s), 1599 (s), 1563 (w), 1511 (w), 1475 (w), 1462 (w), 1405 (w), 1393 (w), 1320 (s, B-O), 1302 (w), 1281 (s), 1247 (vs), 1178 (s), 1159 (w), 1112 (w), 1052 (s), 1021 (s), 1004 (s), 967 (w), 950 (w), 892 (w), 865 (s), 842 (w), 825 (w), 790 (w), 766 (w), 744 (w), 730 (w), 718 (w), 667 (w), 650 (w), 624 (w), 591 (s) cm^{-1}.

*3.3. Tris[4-(dodecyloxy)phenyl]boroxine (**3g**)*

Purification by chromatography on SiO_2 with hexanes/EtOAc (20:1), colorless solid (30%, purity > 95%), m.p. 98 °C, R_f = 0.25. ^{1}H NMR (500 MHz, $CDCl_3$): δ = 0.88 (t, J = 6.9 Hz, 9H, CH_3), 1.19–1.43 (m, 48H, CH_2), 1.42–1.52 (m, 6H, $OCH_2CH_2CH_2$), 1.76–1.87 (m, 6H, OCH_2CH_2), 4.03 (t, J = 6.6 Hz, 6H, OCH_2), 6.99 (d, J = 8.6 Hz, 6H, 3-H), 8.14 (d, J = 8.5 Hz, 6H, 2-H) ppm. ^{13}C NMR (125 MHz, $CDCl_3$): δ = 14.1 (CH_3), 22.7 (CH_2CH_3), 26.1, 29.30, 29.37, 29.43, 29.60, 29.62, 29.65, 29.68, 31.9 (CH_2), 67.9 (OCH_2), 114.0 (C-3), 137.5 (C-2), 162.8 (C-4) ppm. FT-IR (ATR): $\tilde{\nu}$ = 2918 (s), 2849 (s), 1601 (s), 1567 (w), 1512 (w), 1472 (w), 1413 (s), 1351 (s, B-O), 1305 (s), 1288 (s), 1270 (w), 1240 (vs), 1174 (s), 1115 (w), 1030 (w), 1005 (w), 999 (w), 973 (w), 916 (w), 891 (w), 834 (w), 819 (w), 747 (s), 720 (w), 688 (s, BX), 631 (w), 576 (s) cm^{-1}. MS (MALDI-TOF): *m*/*z* calcd. for [$C_{54}H_{87}BO_6$–H^+] 864.67; found 863.57. Elemental analysis calculated for $C_{54}H_{87}B_3O_6$ (864.71 g·mol^{-1}): C 75.01, H 10.14; found: C 75.11, H 10.08.

*3.4. 3,4-Bis(dodecyloxy)phenylboronic Acid (**6c**)*

Purification by chromatography (hexanes/EtOAc, 20:1), colorless solid (11%, purity > 95%), m.p. 67 °C, R_f = 0.3. ^{1}H NMR (300 MHz, $CDCl_3$) δ = 0.88 (m_C, 6H, CH_2CH_3), 1.18–1.54 (m, 28H, CH_2), 1.72–1.91 (m, 4H, OCH_2CH_2), 3.99–4.07 (m, 4H, OCH_2CH_2), 5.67 (s, 2H, OH) 6.90–6.96 (m, 1H, 5-H), 7.32–7.39 (m, 2H, 2-H, 6-H) ppm. ^{13}C NMR (75 MHz, $CDCl_3$) δ = 14.1 (CH_3), 22.7 (CH_2CH_3), 26.06, 26.09, 29.2, 29.38, 29.41, 29.45, 29.49, 29.61, 29.62, 29.66, 29.67, 31.9 (CH_2), 68.9, 69.3 (OCH_2), 112.8 (C-2), 119.8 (C-5), 128.8 (C-6), 148.4, 151.8 (C-3, C-4) ppm. FT-IR (ATR): $\tilde{\nu}$ = 3453 (w, B*O-H*), 2955 (w), 2915 (vs), 2849 (s), 1595 (w), 1515 (w), 1468 (s), 1410 (s), 1334 (s, B-O), 1292 (s), 1254 (s), 1203 (s), 1140 (s), 1095 (w), 1072 (w), 1030 (w), 999 (w), 975 (w), 956 (w), 866 (w), 811 (w), 791 (w), 765 (w), 740 (w), 703 (w), 683 (w), 599 (w), 535 (w) cm^{-1}.

*3.5. 3,4,5-Tris(undecyloxy)phenylboronic Acid (**10d**)*

Purification by chromatography (hexanes/EtOAc, 20:1), colorless solid (11%, purity > 95%), m.p. 53 °C, R_f = 0.3. ^{1}H NMR (500 MHz, $CDCl_3$): δ = 0.84–0.93 (m, 9H, CH_3), 1.19–1.40 (m, 42H, CH_2), 1.40–1.54 (m, 6H, $OCH_2CH_2CH_2$), 1.73–1.85 (m, 6H, OCH_2CH_2), 3.95–4.06 (m, 6H, OCH_2), 5.69 (s, 1H, BOH), 6.99 (s, 2H, H-2) ppm. ^{13}C NMR (126 MHz, $CDCl_3$): δ = 14.1 (CH_3), 22.7 (CH_2CH_3), 26.15, 26.17, 29.39, 29.42, 29.47, 29.50, 29.64, 29.68, 29.69, 29.73, 29.76, 29.78, 30.4, 31.9, 32.0 (CH_2), 69.2, 73.5 (OCH_2), 113.00 (C-2), 131.65 (C-1), 141.06 (C-4), 152.90 (C-3) ppm. FT-IR (ATR): $\tilde{\nu}$ = 3437 (w), 2956 (w), 2917 (s), 2872 (w), 2849 (s), 1572 (w), 1502 (w),

1467 (w), 1407 (s), 1377 (w), 1337 (s, B-O), 1284 (w), 1240 (w), 1199 (w), 1115 (s), 1067 (w), 1002 (w), 972 (w), 943 (w), 888 (w), 845 (w), 831 (w), 703 (w), 640 (w), 607 (w), 586 (w) cm^{-1}. MS (ESI): *m*/*z* = 632 [M–H^+], 604, 472. HRMS (ESI): *m*/*z* calcd. for [$C_{39}H_{73}BO_5$] 631.55; found 631.5453.

*3.6. General Procedure for the Synthesis of Alkoxyphenylboroxines (**5**) [6–9]*

To a solution of the appropriate **4** (2.50 mmol) in abs. THF (50 mL) at −78 °C was added *n*-BuLi (3.13 mL, 5.00 mmol, 1.6 M in n-hexane) and the reaction mixture stirred for 2 h. Then trimethylborate (0.68 mL, 0.63 mg, 6.00 mmol) was added and the reaction mixture stirred for a further 1 h at −78 °C. After warming to room temperature, the reaction was terminated by addition of HCl (25 mL, 2 M Lösung) and stirring for 1 h. The resulting aqueous suspension was extracted with Et_2O (3 × 30 mL). The combined organic layers were washed with H_2O (2 × 20 mL) and brine (30 mL) and dried ($MgSO_4$). The solvent was removed under vacuum and the crude product purified by column chromatography on SiO_2 and/or recrystallization.

*3.7. Tris[3,4-bis(decyloxy)phenyl]boroxine (**5b**)*

Purification by chromatography (hexanes/EtOAc, 20:1) and recrystallization from hexane at room temperature , colorless solid (31%, purity > 95%), m.p. 118 °C, R_f = 0.3 (CH_2Cl_2/Et_2O, 10:1). ^{1}H NMR (500 MHz, $CDCl_3$) δ = 0.88 (m_C, 18H, CH_3), 1.19–1.60 (m, 84H, CH_2), 1.77–1.94 (m, 12H, OCH_2CH_2), 4.08 (t, *J* = 6.6 Hz, 6H, OCH_2CH_2), 4.13 (t, *J* = 6.6 Hz, 6H, OCH_2CH_2), 6.99 (d, *J* = 8.1 Hz, 3H, 5-H), 7.69 (d, *J* = 1.4 Hz, 3H, 2-H), 7.81 (dd, *J* = 8.1, 1.4 Hz, 3H, 6-H) ppm. ^{13}C NMR (125 MHz, $CDCl_3$) δ = 14.1 (CH_3), 22.7 (CH_2CH_3), 26.05, 26.14, 29.2, 29.37, 29.40, 29.45, 29.5, 29.60, 29.64, 29.70, 31.93, 31.94 (CH_2), 68.8, 69.5 (OCH_2), 112.5 (C-2), 120.5 (C-5), 130.0 (C-6), 148.4, 153.3 (C-3, C-4) ppm. FT-IR (ATR): $\tilde{\nu}$ = 2918 (s), 2849 (s), 1599 (w), 1520 (w), 1467 (w), 1413 (s), 1377 (s), 1351 (s, B-O), 1324 (s), 1272 (s), 1254 (vs), 1212 (s), 1199 (s), 1138 (s), 1096 (w), 1071 (w), 1020 (w), 986 (w), 935 (w), 874 (w), 814 (w), 791 (w), 741 (w), 710 (s, BX), 672 (w), 594 (w), 546 (w) cm^{-1}. MS (MALDI-TOF): *m*/*z* calcd. for [$C_{78}H_{135}B_3O_9$–2 H]$^+$ 1249.04; found 1247.89. Elemental analysis calculated for $C_{78}H_{135}B_3O_6$ (1249.36 g·mol^{-1}): C 74.99, H 10.89; found: C 75.11, H 10.89.

*3.8. Tris[3,4-bis(dodecyloxy)phenyl]boroxine (**5c**)*

Recrystallization from pentane at room temperature and acetone at −28 °C, colorless solid (20%, purity > 95%), m.p. 108 °C, R_f = 0.3 (CH_2Cl_2/Et_2O, 10:1). ^{1}H NMR (500 MHz, $CDCl_3$): δ = 0.81 (t, *J* = 6.9 Hz, 18H, CH_3), 1.10–1.52 (m, 108H, CH_2), 1.70–1.87 (m, 12H, OCH_2CH_2), 3.98–4.10 (m, 12H, OCH_2), 6.99 (d, *J* = 8.1 Hz, 3H, 5-H), 7.62 (d, *J* = 1.3 Hz, 3H, 2-H), 7.74 (dd, *J* = 8.1, 1.3 Hz, 3H, 6-H) ppm. ^{13}C NMR (126 MHz, $CDCl_3$): δ = 14.1 (CH_3), 22.7 (CH_2CH_3), 26.1, 26.15, 29.2, 29.39, 29.40, 29.46, 29.5, 29.65, 29.68, 29.71, 29.72, 29.76, 31.9 (CH_2), 68.8 (OCH_2), 69.5 (OCH_2), 112.5 (C-2), 120.5 (C-5), 129.9 (C-6), 148.4, 153.3 (C-3, C-4) ppm. FT-IR (ATR): $\tilde{\nu}$ = 2954 (w), 2917 (s), 2848 (s), 1599 (w), 1520 (w), 1467 (w), 1415 (s), 1352 (s, B-O), 1274 (s), 1257 (vs), 1213 (s), 1138 (s), 1096 (w), 1073 (w), 996 (w), 960 (w), 874 (w), 814 (w), 791 (w), 741 (w),

710 (s, BX), 672 (w), 595 (w), 553 (w) cm^{-1}. MS (MALDI-TOF): *m*/*z* calcd. for $[C_{90}H_{159}B_3O_9–4\ H]^+$ 1417.23; found 1413.34.

*3.9. Tris[3,4-bis(tetradecyloxy)phenyl] boroxine (**5d**)*

Recrystallization from Et_2O at −28 °C, colorless solid (7%, purity > 95%), m.p. 112 °C, R_f = 0.3 (CH_2Cl_2/Et_2O, 10:1). ^{1}H NMR (500 MHz, $CDCl_3$): δ = 0.88 (t, *J* = 6.9 Hz, 18H, CH_3), 1.21–1.59 (m, 132H, CH_2), 1.77–1.93 (m, 12H, OCH_2CH_2), 4.04–4.17 (m, 12H, OCH_2), 6.99 (d, *J* = 8.1 Hz, 3H, 5-H), 7.69 (d, *J* = 1.3 Hz, 3H, 2-H), 7.81 (dd, *J* = 8.1, 1.3 Hz, 3H, 6-H) ppm. ^{13}C NMR (126 MHz, $CDCl_3$): δ = 14.1 (CH_3), 22.7 (CH_2CH_3), 26.1, 26.2, 29.2, 29.4, 29.5, 29.6, 29.66, 29.69, 29.70, 29.72, 29.8, 31.9 (CH_2), 68.8 (OCH_2), 69.5 (OCH_2), 112.5 (C-2), 120.5 (C-5), 129.9 (C-6), 148.4, 153.3 (C-3, C-4) ppm. FT-IR (ATR): $\tilde{\nu}$ = 2954 (w), 2917 (vs), 2848 (s), 1599 (w), 1520 (w), 1467 (w), 1415 (s), 1378 (s), 1352 (s, B-O), 1256 (s), 1212 (s), 1138 (s), 1097 (w), 1074 (w), 1019 (w), 1006 (w), 974 (w), 873 (w), 815 (w), 792 (w), 741 (w), 710 (s, BX), 672 (w), 595 (w) cm^{-1}. MS (MALDI-TOF): *m*/*z* calcd. for $[C_{102}H_{183}B_3O_9 + Na]^+$ 1608.39; found 1604.32. Elemental analysis calculated for $C_{102}H_{183}B_3O_9$ (1586.00 $g\cdot mol^{-1}$): C 77.25, H 11.63; found: C 77.27, H 11.33.

*3.10. 3,4,5-Tris(dodecloxy)phenyl-boro-diethanolamino Complex (**9e**)*

^{1}H-NMR (300 MHz, $CDCl_3$): δ = 0.76–0.98 (m, 9H, CH_3), 1.11–1.54 (m, 54H, CH_2), 1.58–1.95 (m, 6H, OCH_2CH_2), 2.78 (s, 2H, a-H oder b-H), 3.26 (s, 2H, a-H oder b-H), 3.77–4.12 (m, 10H, OCH_2, a-H oder b-H), 4.86 (s, 1H, N-H), 6.72 (s, 2H, 2-H) ppm. ^{13}C-NMR (75 MHz, $CDCl_3$): δ = 14.1 (CH_3), 22.7, 26.2, 29.4, 29.5, 29.7, 30.4, 31.9 (CH_2), 51.5, 63.6 (C-a, C-b), 69.05, 73.39 (OCH_2), 110.51 (C-2), 137.64 (C-4), 152.55 (C-3) ppm.

4. DSC Traces

Figure S3. DSC traces of the boroxines **11** (2nd heating/cooling cycle; heating/cooling rate 5 $K\cdot min^{-1}$).

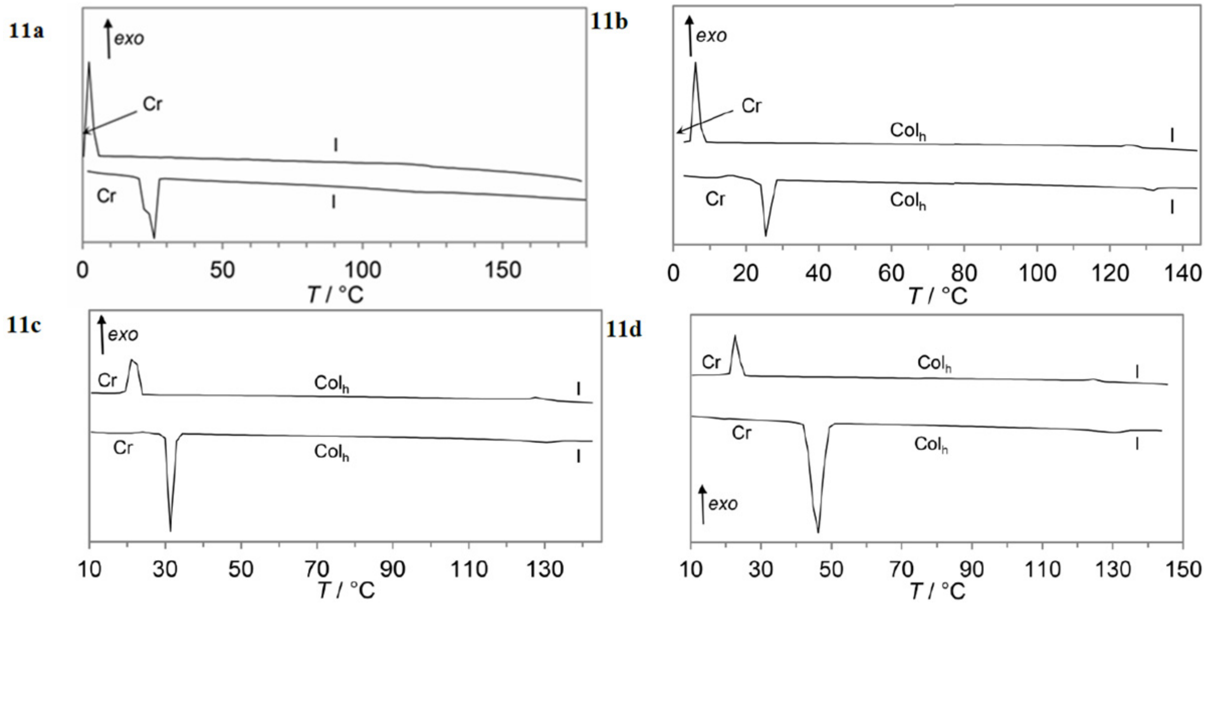

Figure S3. *Cont.*

11e

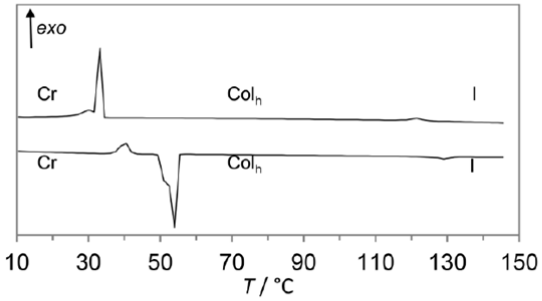

5. XRD Investigations

Figure S4. SAXS profile of the liquid-crystalline Phase of **11** at respective temperatures (Inset: wide-angle scattering, WAXS).

11b
(at 100 °C)

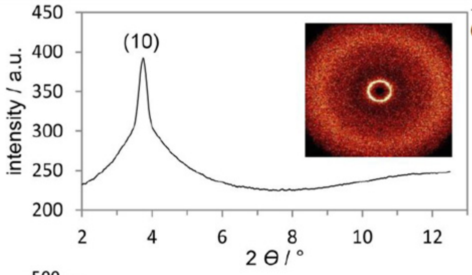

11c
(at 65 °C)

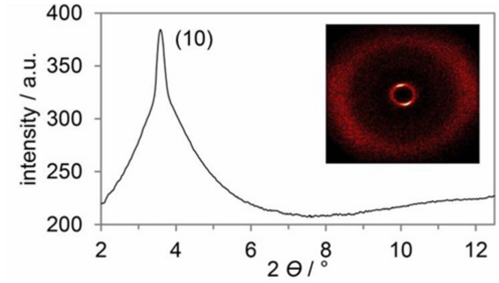

11d
(at 110 °C)

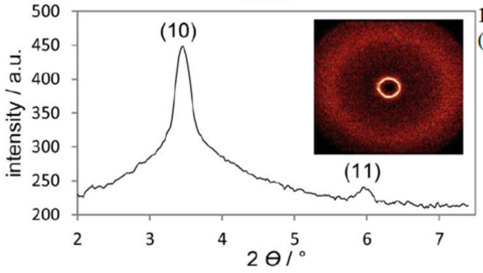

11e
(at 100 °C)

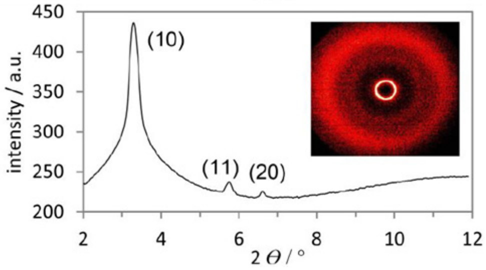

Table S3. XRD results for boroxines **11b–d**.

Compound	Mesophase	Lattice Spacing/Å	*d* spacing/Å Observed (Calcd)	Miller Indices	*Z*
11b	Col$_h$ at 100 °C *p*6 mm	*a* = 27.3	23.6 4.5	(10) (halo)	1.1
11c	Col$_h$ at 65 °C *p*6 mm	*a* = 28.4	24.6 4.7	(10) (halo)	1.2
11d	Col$_h$ at 110 °C *p*6 mm	*a* = 29.5	25.6 14.8 4.6	(10) (11) (halo)	1.1
11e	Col$_h$ at 100 °C *p*6 mm	*a* = 31.0	26.8 15.4 (15.5) 13.3 (13.4) 4.7	(10) (11) (20) (halo)	1.2

6. NMR Data

Figure S5. ^{1}H NMR and ^{13}C NMR spectra of complex **9e**, boronic acid **6c** and boroxines **11a–e**.

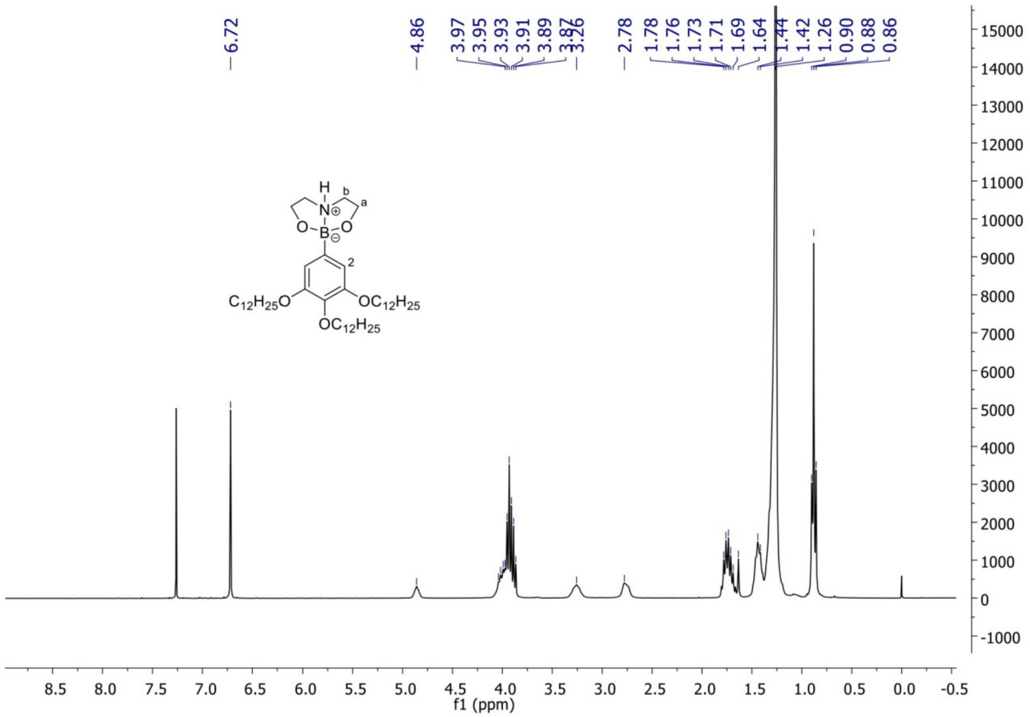

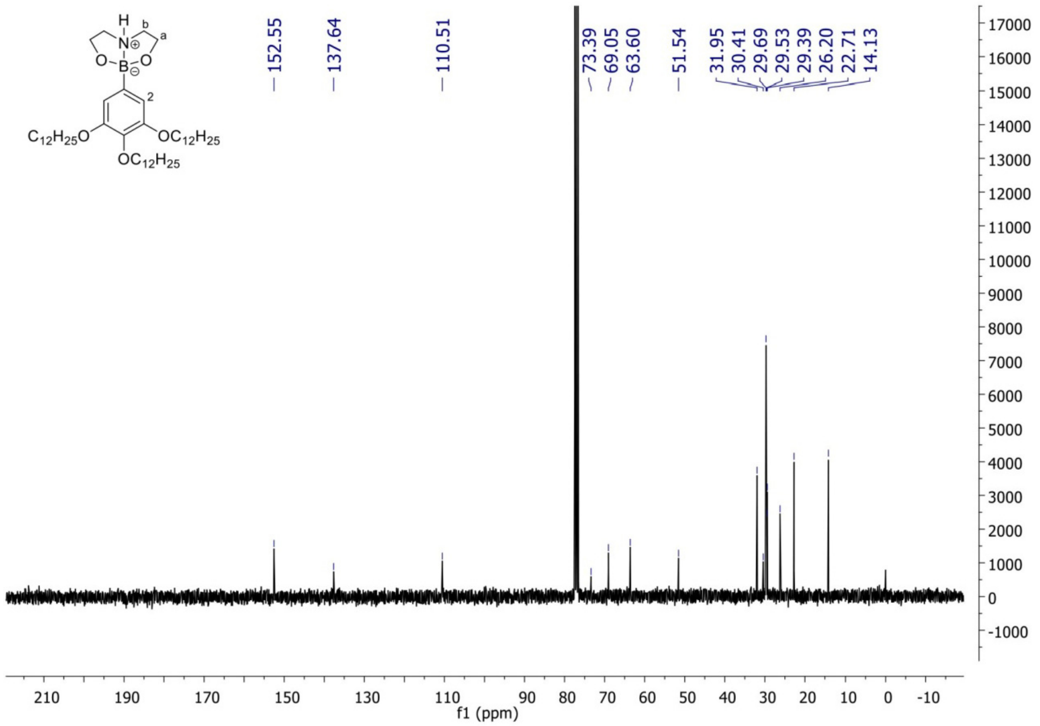

Figure S5. *Cont*.

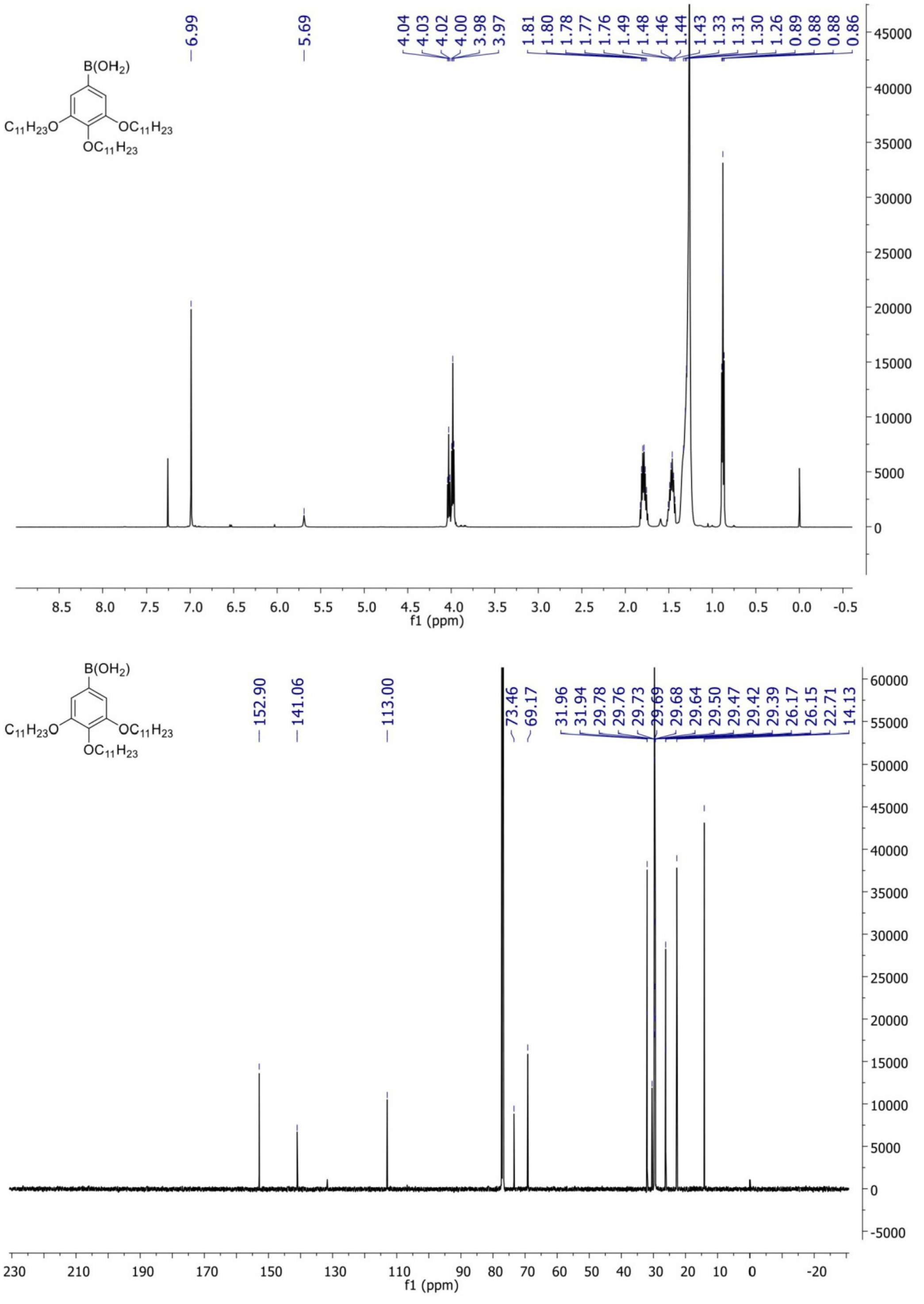

Figure S5. *Cont*.

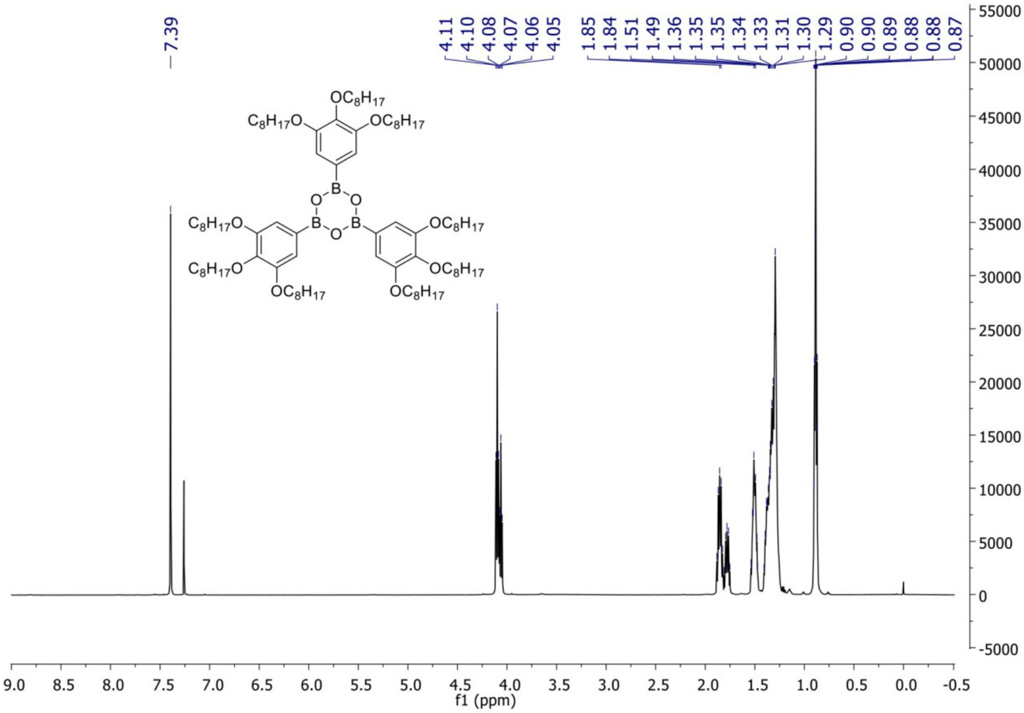

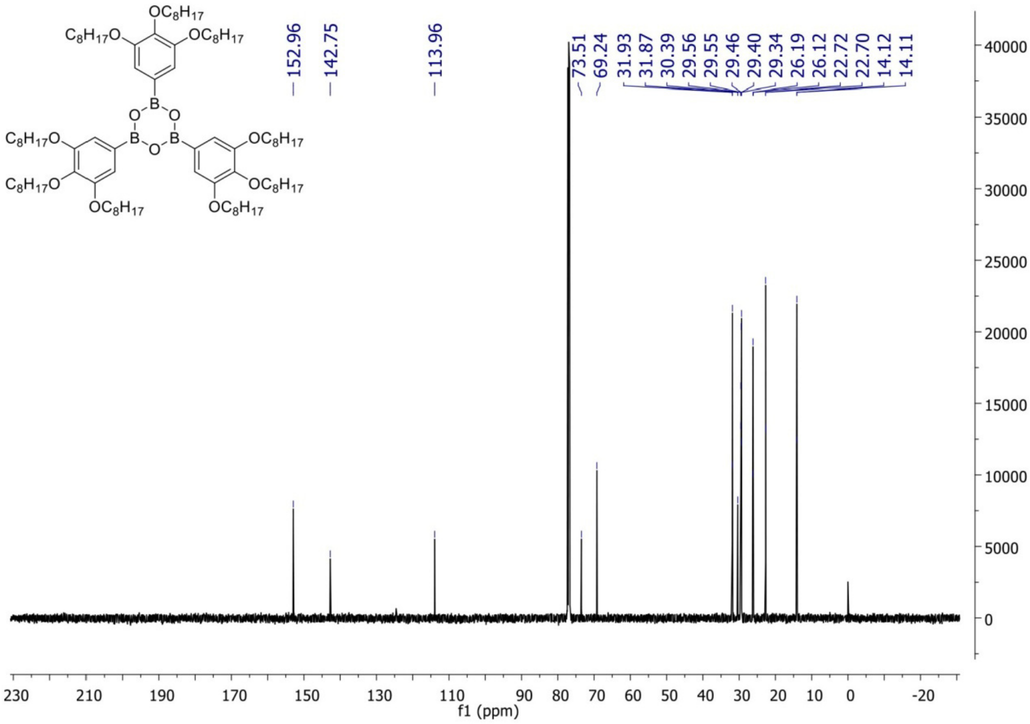

Figure S5. *Cont*.

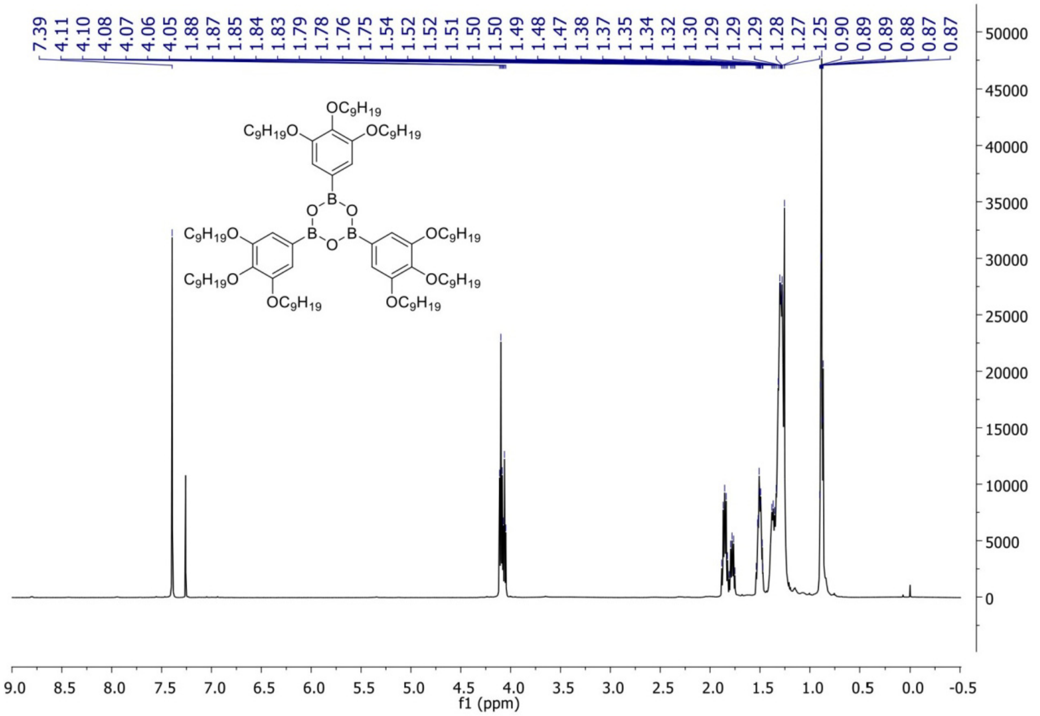

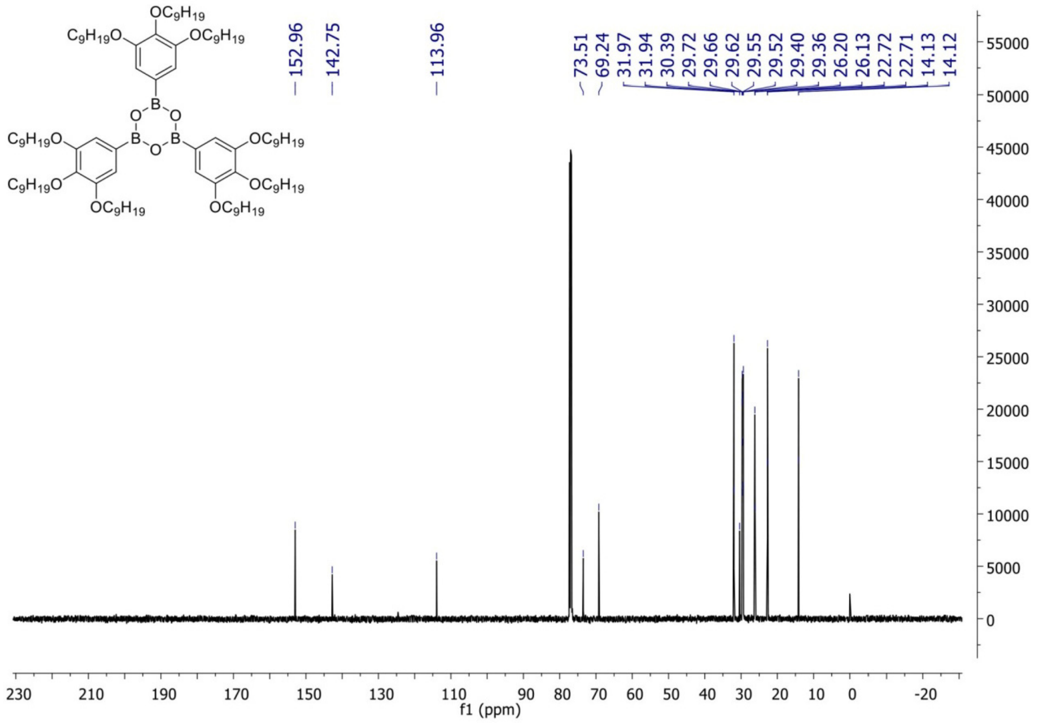

Figure S5. *Cont*.

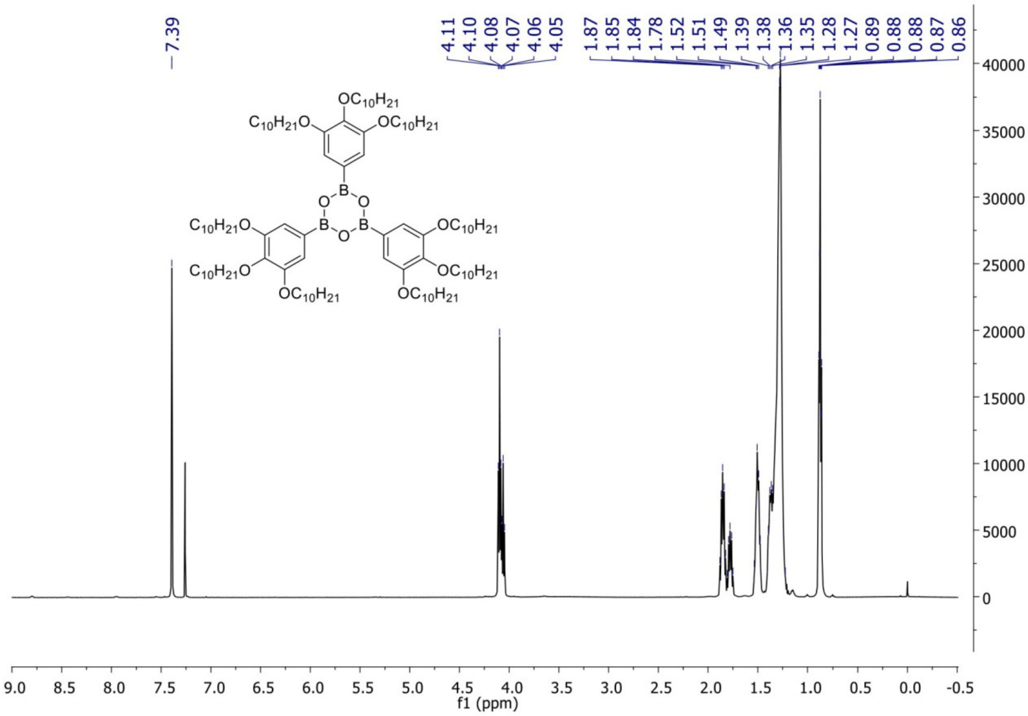

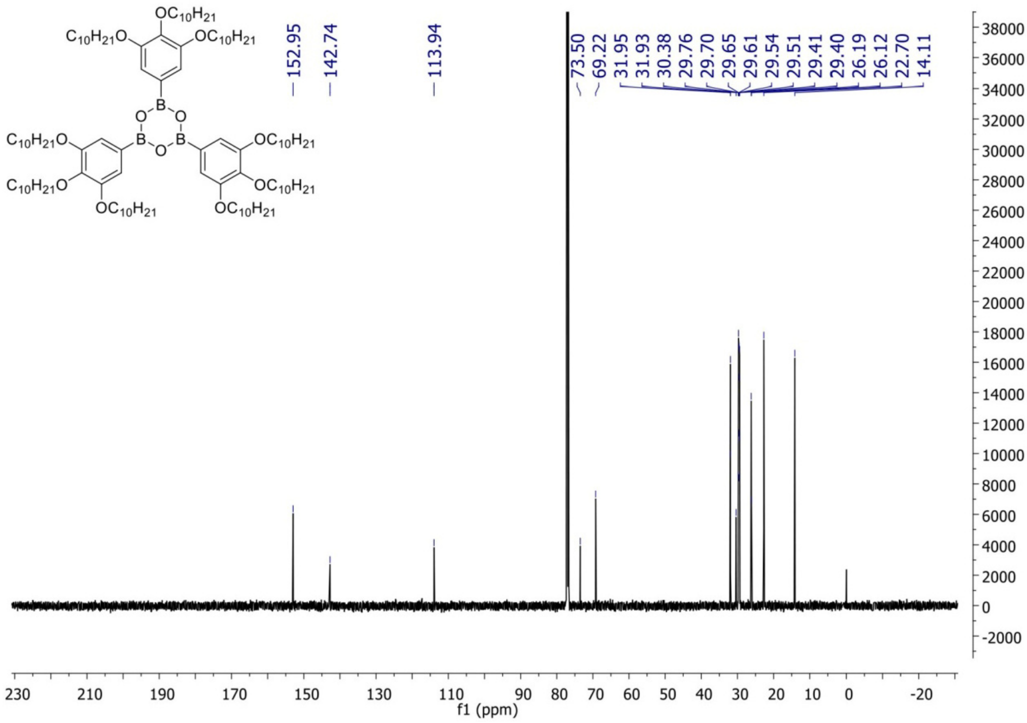

Figure S5. *Cont.*

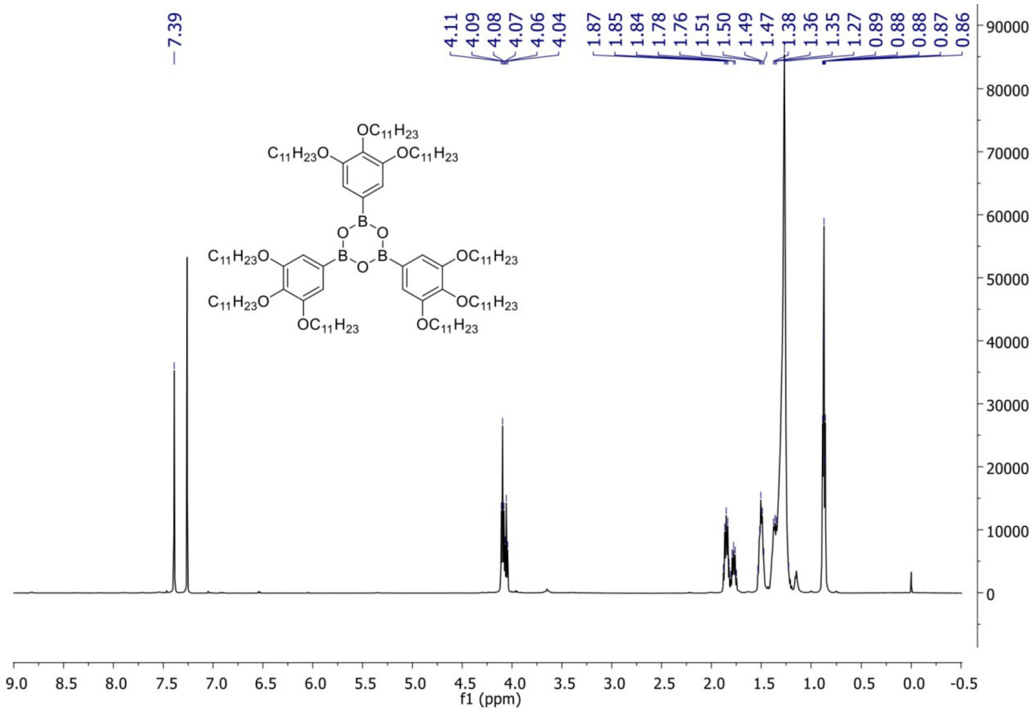

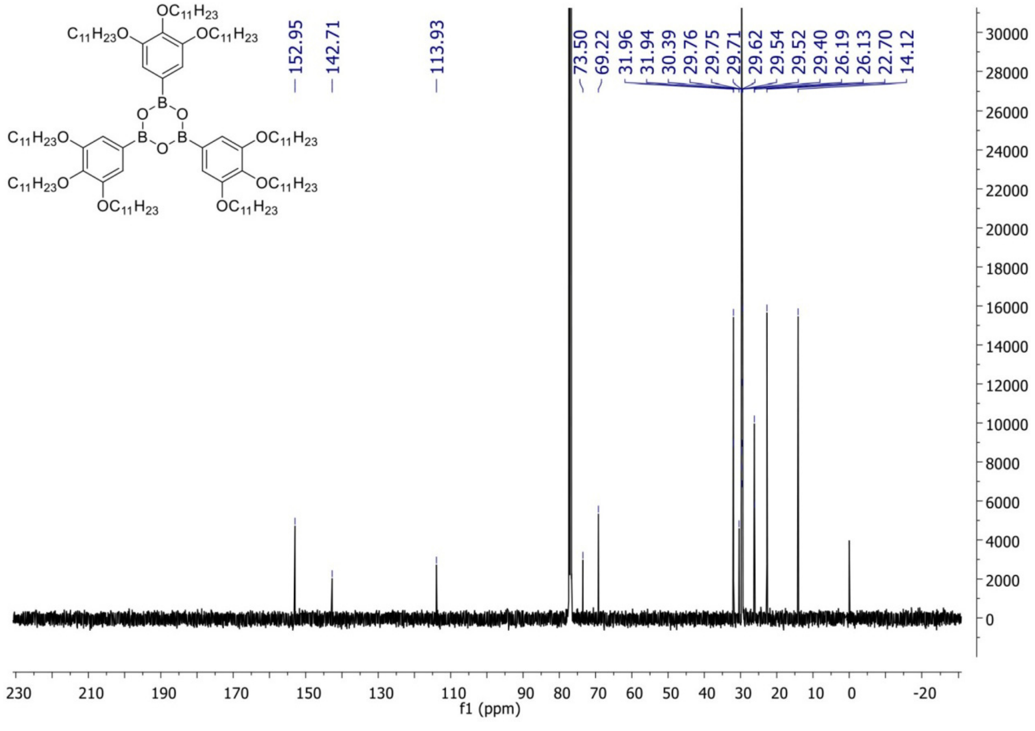

Figure S5. *Cont.*

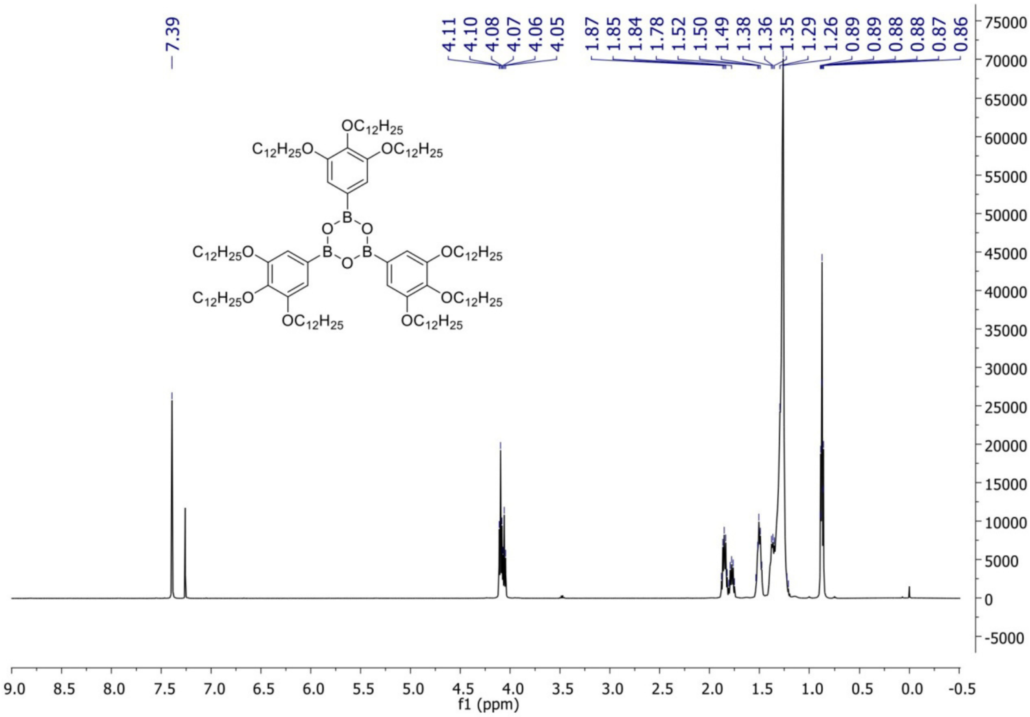

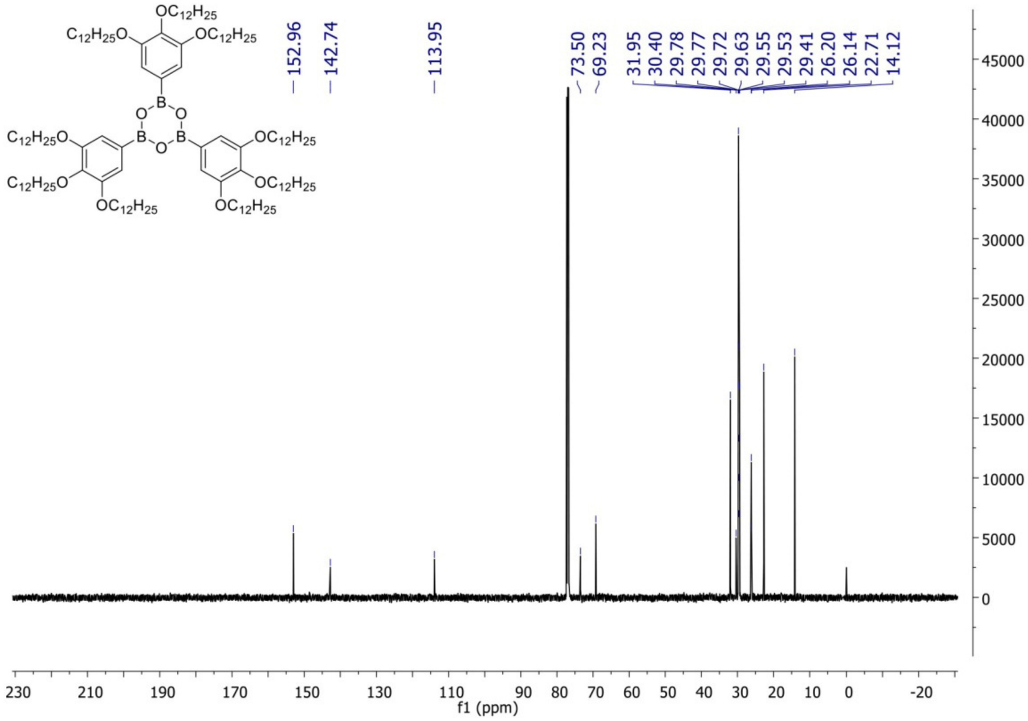

References

1. Wu, X.; Liu, X.; Zhao, G. Catalyzed asymmetric aryl transfer reactions to aldehydes with boroxines as aryl source. *Tetrahedron Asymmetry* **2005**, *16*, 2299–2305.

2. Mansueto, M. Ionische Flüssigkristalle aus Aminosäuren und Arbeiten zum Aufbau von Thiotriphenylenen zur Oberflächenbeschichtung, Ph.D. Thesis, Universität Stuttgart, Stuttgart, Germany, October 2013. (In German)
3. Perttu, E.K.; Arnold, M.; Iovine, P.M. The synthesis and characterization of phenylacetylene tripodal compounds containing boroxine cores. *Tetrahedron Lett.* **2005**, *46*, 8753–8756.
4. Chen, Z.; Mocharla, V.P.; Farmer, J.M.; Pettit, G.R.; Hamel, E.; Pinney, K.G. Preparation of new anti-tubulin ligands through a dual-mode, addition-elimination reaction to a bromo-substituted a,b-unsaturated sulfoxide. *J. Org. Chem.* **2000**, *65*, 8811–8815.
5. Snyder, H.R.; Konecky, M.S.; Lennarz, W.J. Aryl Boronic Acids. II. Aryl Boronic Anhydrides and their Amine Complexes. *J. Am. Chem. Soc.* **1958**, *80*, 3611–3615.
6. Gray, G.W.; Hird, M.; Lacey, D.; Toyne, K.J. The synthesis and transition temperatures of some 4,4″-dialkyl- and 4,4″-alkoxyalkyl-1,1:4′,1″-terphenyls with 2,3- or 2′,3′-difluoro substituents and of their biphenyl analogues. *J. Chem. Soc. Perkin Trans. 2* **1989**, 2041–2053.
7. McElroy, W.T.; DeShong, P. Synthesis of the CD-ring of the anticancer agent streptonigrin: studies of aryl-aryl coupling methodologies. *Tetrahedron* **2006**, *62*, 6945–6954.
8. Schultz, A.; Laschat, S.; Saipa, A.; Gießelmann, F.; Nimtz, M. Schulte, J.L.; Baro, A.; Miehlich, B. Columnar liquid crystals with a central crown ether unit. *Adv. Funct. Mater.* **2004**, *14*, 163–168.
9. Quimby, J.M.; Scott, L.T. Expanding the Suzuki–Heck-type coupling cascade: A new indeno[1,2,3]-annelation of polycyclic aromatic hydrocarbons. *Adv. Synth. Catal.* **2009**, *351*, 1009–1013.
